# The novel isoxazoline ectoparasiticide lotilaner (Credelio™): a non-competitive antagonist specific to invertebrates γ-aminobutyric acid-gated chloride channels (GABACls)

**DOI:** 10.1186/s13071-017-2470-4

**Published:** 2017-11-01

**Authors:** Lucien Rufener, Vanessa Danelli, Daniel Bertrand, Heinz Sager

**Affiliations:** 1 Elanco Animal Health, Mattenstrasse 24a, CH-4058 Basel, Switzerland; 2 HiQScreen Sàrl, Route de Compois 6, CH-1222 Vésenaz, Switzerland

**Keywords:** Lotilaner, γ-aminobutyric acid-gated chloride channels, GABACl, Isoxazoline, Invertebrates, Non-competitive antagonist

## Abstract

**Background:**

The isoxazolines are a novel class of parasiticides that are potent inhibitors of γ-aminobutyric acid (GABA)-gated chloride channels (GABACls) and, to a lesser extent, of inhibitory glutamate-gated chloride channels (GluCls). Lotilaner (Credelio™), a novel representative of this chemical class, is currently evaluated for its excellent ectoparasiticide properties.

**Methods:**

In this study, we investigated the molecular mode of action and pharmacology of lotilaner. We report the successful gene identification, cDNA cloning and functional expression in *Xenopus* oocytes of *Drosohpila melanogaster* (wild type and dieldrin/fipronil-resistant forms), *Lepeophtheirus salmonis* (an ectoparasite copepod crustacean of salmon), *Rhipicephalus microplus* and *Canis lupus familiaris* GABACls. Automated *Xenopus* oocyte two-electrode voltage clamp electrophysiology was used to assess GABACls functionality and to compare ion channel inhibition by lotilaner with that of established insecticides addressing GABACls as targets.

**Results:**

In these assays, we demonstrated that lotilaner is a potent non-competitive antagonist of insects (fly*)* GABACls. No cross-resistance with dieldrin or fipronil resistance mutations was detected, suggesting that lotilaner might bind to a site at least partly different from the one bound by known GABACl blockers. Using co-application experiments, we observed that lotilaner antagonism differs significantly from the classical open channel blocker fipronil. We finally confirmed for the first time that isoxazoline compounds are not only powerful antagonists of GABACls of acari (ticks) but also of crustaceans (sea lice), while no activity on a dog GABA_A_ receptor was observed up to a concentration of 10 μM.

**Conclusions:**

Together, these results demonstrate that lotilaner is a non-competitive antagonist specific to invertebrate’s γ-aminobutyric acid-gated chloride channels (GABACls). They contribute to our understanding of the mode of action of this new ectoparasiticide compound.

**Electronic supplementary material:**

The online version of this article (10.1186/s13071-017-2470-4) contains supplementary material, which is available to authorized users.

## Background

**Fig. 1:**
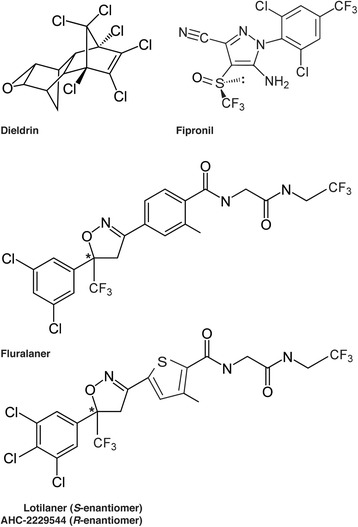
Chemical structures of chloride channel antagonists. The star indicates the chiral center for both isoxazoline molecules. The biologically active compound lotilaner is the *S*-enantiomer while the *R*-enantiomer (AHC-2229544) is inactive. Fluralaner is a racemic mixture containing both enantiomers

**Fig. 2:**
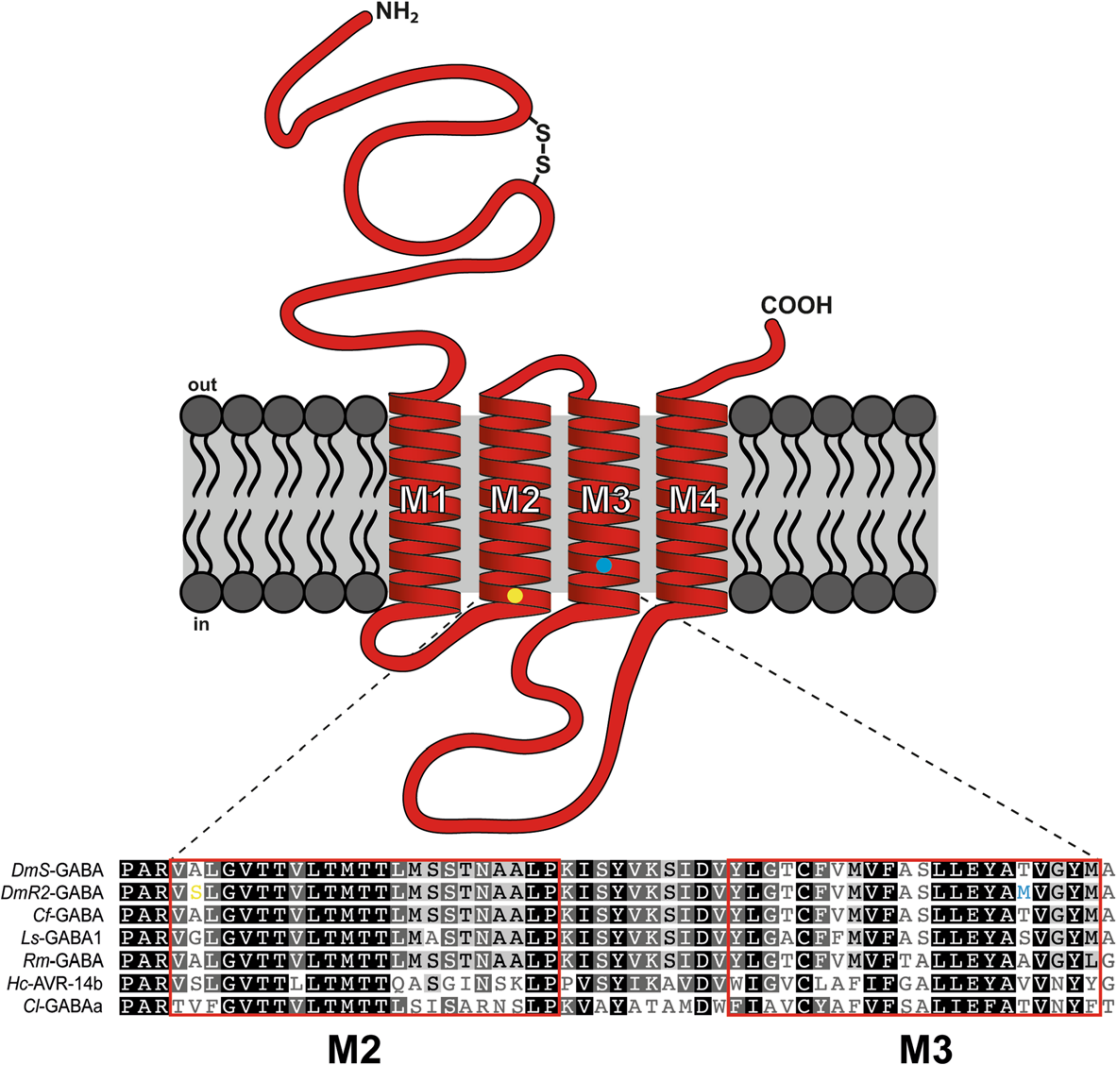
Schematic representation of a ligand-gated ion channel subunit. Location of the residues mutated in the transmembrane domains M2 and M3 that have shown to confer resistance to dieldrin and fipronil are represented by a yellow and blue circle respectively. Motifs typical for Cys-loop LGIC such as a large N-terminal extracellular domain, four transmembrane domains (M1-M4), an intracellular loop connecting the M3 and M4 segments and a Cys-loop (2 cysteines) are visible. The aligned amino acid sequences from different species (Dm, *Drosophila melanogaster*; Cf*, Ctenocephalides felis*; Ls, *Lepeophtheirus salmonis*; Rm, *Rhipicephalus microplus*; Hc, *Haemonchus contortus*; Cl, *Canis lupus familiaris*) show that the M2 to M3 region is highly conserved even between different phyla. Both mutations A301S and T350 M are highlighted in yellow and blue, respectively, in the DmR2-GABA sequence

Neurotransmitter receptors are membrane proteins that are directly involved in transmembrane signalling in both neurons and muscle cells. They are important for the function and regulation of the nervous system but also as common targets of drugs, endo- and ecto-parasiticides [1]. Phenylpyrazoles such as fipronil (Fig. 1) and ethiprole, and macrolides such as avermectins and milbemycins, are commercially available insecticides and parasiticides that target γ-aminobutyric acid (GABA)-gated chloride channels (GABACls) and inhibitory glutamate-gated chloride channels (GluCls) in invertebrates [2]. The GABACls are members of the Cys-loop ligand-gated ion channel (LGIC) family, together with GluCls, nAChRs and glycine receptors. Members of this family have several characteristically conserved motifs such as a large N-terminal extracellular domain, four transmembrane domains (M1-M4), and a long, variable intracellular loop connecting the M3 and M4 segments within each subunit. The N-terminal extracellular domain contains a cysteine disulfide loop (Fig. 2). Five subunits form an integral chloride channel at the centre, with the M2 domain arranged toward the channel pore. The endogenous agonist-binding site resides in the extracellular interface between the N-terminal domains of two adjacent subunits; this extracellular interface is called the orthosteric site.

GABA receptors were first shown to be a target of the organochlorine insecticides dieldrin (Fig. 1) and lindane [3, 4], both of which were banned because of their environmental persistence. In the past three decades, structurally diverse insecticidal compounds have been reported to act as non-competitive antagonists or blockers for GABA and inhibitory glutamate receptors [2]. No further development of new classes of insecticides have been reported since the commercialization of the phenylpyrazole (in 1993) and the natural product spinosad (in 1997), two decades ago [5, 6].

Extensive use of these classes for pest control in agriculture and animal health has inevitably led to the selection of drug resistance in targeted parasites. The GABACl, originating from insects with resistance to dieldrin have been intensively studied [7]. These resistant insects have a point mutation (alanine to another amino acid) at around the 300th position in the amino acid sequences of their GABACl subunits. The target gene was named “RDL” for Resistance to DieLdrin (later referred to Dm-GABA in the present report). The RDL derived from *Drosophila melanogaster* contains a mutation of alanine to serine at position 301 (A301S; Fig. 2) [8]. The *Drosophila* GABACls with this mutation shows low sensitivity to dieldrin [9]. Lately, a novel RDL-type mutation was identified in the fipronil-resistant populations of the small brown planthopper, *Laodelphax striatellus* [10]. According to the report, fipronil-resistant *L. striatellus* had an RDL-type mutation (A283N). In addition, Le Goff et al. [11] reported that another RDL-type GABACl of *D. simulans* with two mutations (A301S and T350 M; Fig. 2) showed less sensitivity to fipronil.

A new class of antiparasiticide compounds has been recently discovered containing the compounds fluralaner (A1443; Fig. 1), afoxolaner and sarolaner [12, 13]. Different studies have shown that isoxazolines act as specific blockers of GABACls and to a lesser extent of GluCls channels of insects [14–16]. Since lotilaner (Fig. 1) belongs to the same class, we set out to determine if the molecular mode of action of lotilaner was conserved and whether binding sites were shared with the well-known GABACls blockers (e.g. dieldrin or fipronil). It is interesting to note that fluralaner has a chiral centre and therefore consists of two enantiomers (*S* and *R*). A similar situation is true for lotilaner (Fig. 1), which forms the *S*-enantiomer that has been shown to be active in vivo, while the *R*-enantiomer (AHC-2229544; Fig. 1) shows 100× less biological activity (data not shown). The *R*-enantiomer is removed from the commercially available product Credelio™, which contains only the active *S*-enantiomer (lotilaner).

In this study, we investigate the ectoparasiticidal activity and parasite molecular target pharmacology of lotilaner. We report the successful gene identification, cloning and functional expression in *Xenopus* oocytes of *Lepeophtheirus salmonis* (an ectoparasitic copepod crustacean of salmon) GABACl subunit (Ls-GABA1). Furthermore, *D. melanogaster* (insect) GABACl subunit genes were prepared as wild type and dieldrin/fipronil-resistant forms (DmS-GABA and DmR2-GABA respectively) in addition to *Rhipicephalus microplus* (Acari) and *Canis lupus familiaris* (Beagle breed) GABACls (Rm-GABA and Cl-GABA_A_ α1β2γ2, respectively). For all five GABACls, automated *Xenopus* oocyte two-electrode voltage clamp (TEVC) electrophysiology ion channel assays were used to assess receptor functionality. With these assays, the precise pharmacology of insect, acarine, crustacean and mammalian GABACls were established for their natural agonist GABA, as well as for the antagonists dieldrin and fipronil in comparison with the novel new drug compound lotilaner.

## Methods

### Chemicals

Dieldrin was purchased from Sigma-Aldrich (Buchs, Switzerland) while fipronil, lotilaner and the AHC-2229544 (R-enantiomer) were provided by Elanco Animal Health Inc. Switzerland. The chiral purity of lotilaner and AHC-2229544 was 99.9 and 99.65%, respectively. The modulatory compounds were prepared as 10 mM stock solutions in dimethyl sulfoxide (DMSO) and were dissolved in oocyte Ringer’s OR2 medium (see below), resulting in a maximal final DMSO concentration of 0.1%. GABA was obtained from Sigma-Aldrich and prepared as stock solution at 100 mM in ddH_2_O.

### Cloning of GABACls cDNA

A total of 5 GABACls subunits was cloned and expressed in *Xenopus* oocytes. For the cloning of the sea lice (Ls-GABA1) and the dog (Cl-GABA_A_ α1β2γ2) GABACls, RNA extraction, cDNA synthesis and PCR amplification were performed using previously described protocols [17]. For Ls-GABA1, total RNA was extracted from one entire male sea louse parasite from which, 1 μg of total RNA (DNase-treated) was reverse-transcribed to cDNA using a (dT)30 primer and SuperScript III Reverse Transcriptase (Invitrogen, Carlsbad, CA, USA) and the First choice RLM-RACE Kit from Ambion (AM1700, Waltham, MA, USA). Gene-specific primers (Additional file 1: Table S1) were designed using the Primer3 software (available at http://www.bioinfo.ut.ee/primer3-0.4.0//). A rapid amplification of cDNA-ends by PCR (RACE-PCR) was performed using internal reverse primers Ls-GABA1_R6 and Ls-GABA1_R7 combined with the 5′ RACE Outer and Inner Primer (from the Ambion kit) to obtain the 5′-untranslated region (UTR). Internal forward primers Ls-GABA1_F2 and Ls-GABA1_F3 combined with a poly(dT) primer were used for the 3′-UTR of the transcript (Supplementary Table S1). Start and stop codons were deduced from the 5′ and 3′-RACE product sequences. The gene-specific PCR to obtain the full-length Ls-GABA1 from *L. salmonis* cDNA was performed with a Phusion polymerase (New England Biolabs, Ipswich, MA, USA) and primer pair NheI_Ls-GABA1_F1 and SpeI_Ls-GABA1_R1 (Additional file 1: Table S1). The reaction conditions were: 98 °C for 30 s; 32 cycles of (98 °C for 10 s; 60 °C for 20 s; 72 °C for 45 s); 72 °C for 10 min. For Cl-GABA_A_ α1β2γ2 subunits, total RNA was extracted from a piece of dog brain (Beagle breed) and cDNA synthetized as described above. The brain sample was obtained from a non-infected control dog of an efficacy-study run at the Centre de Recherche Santé Animale SA (approved by the Cantonal Veterinary Authorities of Fribourg, permit number N° 2010_46_FR). The following primers were used to amplify full-length coding sequences based on published sequences (GenBank accessions XM_546261.5, XM_014113040.1 and XM_546259.5). For Cl-GABAa1: NheI_Cl-GABAa1_F1 and XhoI_Cl-GABAa1_R1. For Cl-GABAb2: NheI_Cl-GABAb2_F1 and XhoI_Cl-GABAb2_R1. For Cl-GABAg2: NheI_Cl-GABAg2_F1 and XhoI_Cl-GABAg2_R1 (Additional file 1: Table S1). The reaction conditions were the same as for Ls-GABA1. PCR products were analyzed on 1% agarose gels, excised, gel-purified using a NucleoSpin kit (Macherey Nagel, Düren, Germany), and cloned into pJET1.2 using the Thermo Scientific CloneJET PCR kit (catalog N° K1231, Waltham, MA, USA). Plasmid DNA was purified using the QIAprep Spin Miniprep Kit (Qiagen, Valencia, CA, USA) and at least three clones of each construct were sequenced using the provided pJET1.2 forward and reverse primers at Microsynth (http://www.microsynth.ch). Sequence quality check and assembly was performed using Geneious v5.6.7 [18] and a nucleotide blast was made on-line (NCBI) against the nucleotide collection (nt). The selected inserts were subcloned into a pT7-TS transcription vector (that introduces *X. laevis* b-globin untranslated cDNA to the 5′ and 3′ end of the gene) via the restriction sites inserted in the primers (Additional file 1: Table S1). Plasmid DNA was purified with an EndoFree Plasmid Purification kit (Qiagen). The sequences have been given the following GenBank accession number: KY550371 for Ls-GABA1, KY550368 for Cl-GABAa1, KY550369 for Cl-GABAb2 and KY550370 for Cl-GABAg2.

For the fly (DmS-GABA and DmR2-GABA) and tick (Rm-GABA) GABACls, the corresponding subunits were synthetized at Genewiz (https://www.genewiz.com) based on publically available sequences with NheI and XhoI restriction site inserted at 5′ and 3′ end of each gene (reference sequences GenBank accession numbers: DmS-GABA, NM_168321.3; Rm-GABA, GQ398111.1). For DmR2-GABA, two SNPs have been introduced to create two amino acid substitutions in M2 and M3. Two silent mutations were introduced in the Rm-GABA sequence to get rid of NheI and XhoI internal restriction sites. For both *Drosophila* GABACls, the splice variant RDL*ac* was used [19]. Subcloning and cRNA synthesis were performed as described above. The sequences have been given the following GenBank accession number: KY550372 for DmS-GABA, KY550373 for DmR2-GABA and KY550374 for Rm-GABA.

### Expression of GABACls in *Xenopus laevis* oocytes

Capped cRNAs were synthesized (T7 mMessage mMachine kit, Ambion, Austin, TX, USA) from the linearized vectors containing the different subunits according to the manufacturer’s protocol. cRNA samples were stored at -80 °C until use. Oocytes were prepared and injected using standard procedures [20]. Briefly, ovaries were harvested from *Xenopus* females that were deeply anesthetized by cooling down at 4 °C and with exposure to tricaine mesylate (3-aminobenzoic acid ethyl ester, methane sulfonate salt, 150 mg/l). The animal care of *Xenopus laevis* (accreditation de l’animalerie HiQScreen N° 171) and sacrifice was done according to the guidance set by the veterinary authorities of the canton of Geneva based on art.18 on the animal welfare legislation (LPA, art. 141 Ordonnance sur la protection des animaux, OPAn) with the authorization N° 27479 GE/15/16). Small pieces of ovary were isolated in a sterile Barth solution containing: NaCl (88 mM), KCl (1 mM), NaHCO_3_ (2.4 mM), HEPES (10 mM, pH 7.5), MgSO_4_·7H_2_O (0.82 mM), Ca(NO3)_2_·4H_2_O (0.33 mM), CaCl_2_·6H_2_O (0.41 mM), at pH 7.4, and supplemented with 20 μg/ml of kanamycin, 100 U/ml penicillin and 100 μg/ml streptomycin. Oocytes were microinjected using a Roboinject automatic injection system (Multi Channel Systems, Reutlingen, Germany) with 15–25 nl of cRNA solution (5–50 ng/μl) and then incubated at 18 °C in sterile filtered Barth solution. Recordings were made 1–6 days post-cRNA injection.

### Two-electrode voltage-clamp measurements using the HiClamp

Oocytes were impaled with two electrodes filled with 3 M KCl, and their membrane potentials were maintained at -80 mV throughout the experiment. Currents evoked by GABA or drugs were recorded using an automated process equipped with standard two-electrode voltage-clamp configuration (HiClamp, MultiChannel Systems). The principle of this system differs from standard electrophysiology because, instead of applying the compound in the perfusion, the oocyte is moved into a well from a 96-well microtiter plate containing the desired solution. Data were filtered at 10 Hz, captured at 100 Hz and analysed using proprietary data acquisition and analysis software running under Matlab (Mathworks Inc., Natick, MA, USA). Additional analyses were performed in Excel (Microsoft, Redmond, WA, USA). Plots of the peak inward currents as a function of the logarithm of the agonist concentration yield classical concentration-activation and concentration-inhibition curves were readily fitted by single Hill equations. Concentration-activation curves were fitted with the equation:

1 $$ Y=\frac{100}{1+{10}^{H\left( logEC50-X\right)}} $$

where Y is the normalized response, logEC_50_ is the logarithm of the concentration of agonist eliciting half-maximal current amplitude, X is the log of dose or concentration, and *H* is the slope factor or Hill slope. The same equation was used for concentration-inhibition curves but logEC_50_ was replaced by logIC_50_. For Fig. 5, the four parameter Hill equation was used:

2 $$ Y=\frac{\mathit{\operatorname{Min}}+\left(\mathit{\operatorname{Max}}-\mathit{\operatorname{Min}}\right)}{1+{10}^{H\left( logEC50-X\right)}} $$

where Max is the maximal response and Min is the response at the lowest drug concentration. EC_50_ and IC_50_ values were determined from the mean of at least 3 or more cells. Oocytes were washed with oocyte Ringer’s OR2 medium (82.5 mM NaCl, 2.5 mM KCl, 5 mM HEPES, 1.8 mM CaCl_2_·2H_2_O, and 1.8 mM MgCl_2_·6H_2_O, pH 7.4) and experiments carried out at 20 °C.

### Drug applications

Dose-response curves to the natural agonist were obtained by sequential applications for 20 s of increasing concentrations of GABA to oocytes expressing one of the five subunits described above. In experiments in which the channel was challenged several times by GABA, enough time was allowed between applications for the channel to recover from desensitization. To assess antagonist properties, oocytes transfected with the previously described subunits were sequentially pre-exposed for 75 s to the tested compound at 1 nM, 10 nM, 30 nM, 100 nM, 300 nM, 1 μM, 3 μM and 10 μM. After each exposure, the compounds were co-applied for 20 s with GABA at concentrations near the EC_50_ (2 μM for Cl-GABA_A_ α1β2γ2; 10 μM for DmS-GABA and DmR2-GABA; 50 μM for Rm-GABA; and 500 μM for Ls-GABA1). The agonist and the drug were then washed off for 15 s and the oocyte exposed again to the same drug concentration for 15 s before increasing to the next concentration. To establish a baseline response, GABA was initially applied 3 times for 20 s every 1.5 min at the beginning of the experiment.

To further characterize the mode of action of lotilaner, we used a co-application protocol. Oocytes expressing the DmS- or DmR2-GABA receptor were exposed for 30 s to five consecutive GABA (1 μM) applications at 1 min interval to reach a stable baseline. For the next two to five applications, GABA (1 μM) and the drug (100 nM) were co-applied for 30 s again at 1 min interval. Peak currents (I_max_) as well as tail currents (measured after 30 s application) were measured for the first and second co-applications and normalized to currents measured after the fifth GABA application.

GABA concentration-response relationships for DmS-GABA in the presence of 0.1 and 1 μM lotilaner were generated by first applying a control 100 μM GABA for 20 s (used for the normalization), followed by a 1.5 min pre-application of lotilaner, and 20 s applications of 1, 3, 10, 30, 100 and 300 μM GABA in the continued presence of lotilaner with intermediate washes for 30 s. Statistical comparisons were done using unpaired Student’s t test. A *P*-value < 0.05 was considered significant.

### Translation

French translation of the Abstract is available in Additional file 2.

## Results

### Identification and cloning of GABACl subunits

For the identification of the full-length GABACl subunit gene of *L. salmonis* (Ls-GABA1), internal primers were designed based on a published partial sequence (ABI95854.1). The missing cDNA sequences were obtained by 5′- and 3′-RACE using total RNA from a single female parasite as template. Based on the deduced start and stop codon positions in the 5′- and 3′-RACE product sequences of Ls-GABA1, PCR primers were then designed for the PCR amplification of the full-length gene from *L. salmonis* cDNA. PCR error-free version of Ls-GABA1 was cloned into the pT7-TS transcription vector. The deduced polypeptide sequence showed many of the elements typical of the ligand-gated ion channel superfamily. First, the polypeptide possessed the conserved cysteines required for the formation of the cysteine loop, the hallmark of the protein family. Secondly, prediction of transmembrane helices with TMHMM 2.0 showed the presence of four transmembrane domains, with the arrangement of extracellular and intracellular domains in agreement with the known architecture of ligand-gated ion channels (Fig. 2). In the case of *C. lupus familiaris*, the genes encoding alpha, beta and gamma GABACl subunits were amplified from dog total cDNA using full length primer pairs derived from published predicted sequences (XM_546261.5, XM_014113040.1 and XM_546259.5). PCR error-free versions of the three subunits were selected and cloned into the pT7-TS. The genes encoding wild type or dieldrin- and fipronil-resistant *D. melanogaster* GABACl subunits (DmS-GABA and DmR2-GABA respectively) as well as *R. microplus* GABACl, were synthetized using a private company (Genwize, South Plainfield, NJ, USA). RDL receptor subunits in *D. melanogaster* can occur as a variety of different splice variants, resulting in various agonist sensitivities [8, 19]. The alternatively spliced regions lie in exon 3 and 6. These alternative transcripts are named *a*, *b* (exon 3), *c* and *d* (exon 6) and the RDL*ac* variant is considered as the canonical isoform with the highest GABA affinity [8]. As a consequence, both *D. melanogaster* GABACls used in the present study were RDL*ac* variants.

### Functional expression in *Xenopus laevis* oocytes

The functionality of all GABACl subunit genes identified and isolated in this study was investigated by TEVC studies on *Xenopus* oocytes injected with in vitro-transcribed and capped cRNA. The application of GABA resulted in currents > 5 μA, demonstrating that the different subunits were assembled into functional receptors. Individual concentration-response curves with GABA as agonist obtained from oocytes expressing DmS-GABA, DmR2-GABA, Ls-GABA1, Rm-GABA and Cl-GABA_A_ α1β2γ2 are shown in Additional file 3 Figure S1a-e. Each curve was fitted to the Eq. (1) and normalized to the fitted maximal current amplitude. The averaged EC_50_ for GABA ranged from 1.80 ± 0.14 μM (Cl-GABA_A_ α1β2γ2, *n* = 12) to 392.54 ± 11.05 μM (Ls-GABA1, *n* = 20). Rm-GABA showed an intermediate value of 49.96 ± 0.76 (*n* = 10; Table 1 and Additional file 3: Figure S1f). The Hill coefficients were higher than 1 for the five receptors (Table 1) indicating the presence of more than one agonist binding site per receptor. These values were obtained from peak current amplitudes. No obvious difference in traces shape or kinetic where observed between the DmS- and DmR2-GABA receptors and they shared a very similar EC_50_ for GABA (10.52 ± 0.70 μM, *n* = 6 and 12.43 ± 0.54 μM, *n* = 7, respectively; Table 1 and Additional file 3: Figure S1f). Those observations demonstrate that both mutations present in DmR2-GABA do not affect its normal function. All four invertebrate receptors had a slow desensitization under GABA stimulation while the traces recorded from the dog receptor were characterized by a strong and fast desensitization followed by a steady-state current (Additional file 3: Figure S1e).

**Table 1:** GABACl receptors response profiles. EC_50_ in μM ± SEM obtained with the natural agonist GABA

Receptor	Agonist	EC_50_ (μM)	n_H_	*N*
DmS-GABA	GABA	10.52 ± 0.70	1.57 ± 0.15	6
DmR2-GABA	GABA	12.43 ± 0.54	1.51 ± 0.09	7
Ls-GABA1	GABA	392.54 ± 11.05	1.35 ± 0.04	20
Rm-GABA	GABA	49.96 ± 0.76	2.27 ± 0.19	10
Cl-GABA_A_ α1β2γ2	GABA	1.80 ± 0.14	1.31 ± 0.12	12

*Abbreviations*: Dm, *Drosophila melanogaster*; Ls, *Lepeophtheirus salmonis*; Rm, *Rhipicephalus microplus*; Cl, *Canis lupus familiaris*. The Hill coefficient (n_H_) is indicated as well as the number of oocytes evaluated (*N*)

### Lotilaner is a potent antagonist of insects GABA receptors

**Fig. 3:**
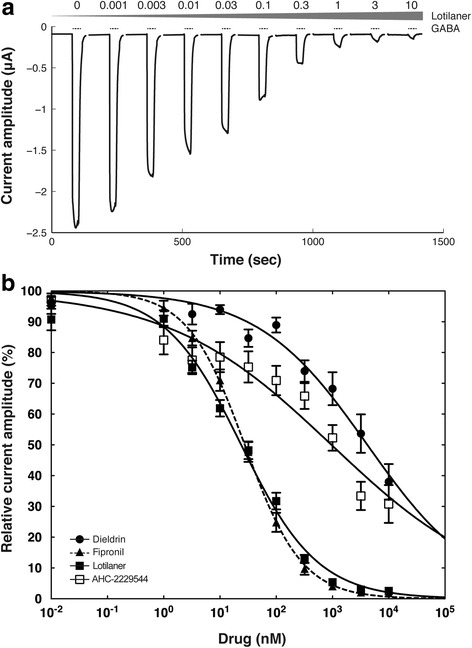
Lotilaner is a potent antagonist of the DmS-GABA receptor. **a** Current traces from a cumulative exposure to increasing dosage of lotilaner obtained for a *Xenopus* oocyte expressing DmS-GABA. The bars indicate the time period of GABA (10 μM) application. The grey triangle represents the gradual exposure to lotilaner with the respective concentration in μM indicated above. **b** Averaged inhibition concentration-response curves measured for dieldrin (black circle), fipronil (black triangle, dashed line), lotilaner (black square), and AHC-2229544 (white square) obtained from oocytes expressing DmS-GABA. Individual curves were standardized to the fitted maximal current amplitude and subsequently averaged. Mean ± SEM of experiments carried out with at least four oocytes from two batches each is shown

Previous studies on isoxazoline derivatives (e.g. fluarlaner) have shown that members of this class were potent inhibitors of insect ligand-gated chloride channels [21]. Picrotoxin, a plant-derived toxin, cyclodiene (dieldrin) or phenylpyrazole (fipronil) insecticides do not bind significantly to unliganded chloride channels and require channel activation to achieve inhibition [7, 11]. Thus, in the present study, a protocol causing a cumulative exposure to the antagonists was used where the oocytes were pre-incubated with the inhibitors for 1.5 min followed by co-application of the inhibitors and GABA to ensure the maximum inhibitory effect would be produced. In a first step, we investigated the effect of lotilaner on the very well characterized *D. melanogaster* GABACl. Figure 3a shows typical traces measured from an oocyte expressing DmS-GABA receptors sequentially exposed to increasing concentration of lotilaner and challenged repeatedly with GABA (10 μM). A strong inhibitory effect was observed with almost complete GABA current inhibition at lotilaner concentrations > 1 μM. Averaged inhibitory concentration-response curves (fitted with Eq. 1) with dieldrin (closed circles), fipronil (closed triangles), lotilaner (closed squares) or AHC-2229544 (open squares) as antagonists are shown in Fig. 3b for DmS-GABA. Dieldrin was found to be a weak inhibitor of DmS-GABA while fipronil proved to be a much more potent inhibitor of this receptor. For dieldrin, the curve was characterized by an IC_50_ of 4170 ± 920 nM and a Hill coefficient of -0.45 ± 0.05 (mean ± SEM, *n* = 9, Table 2). For fipronil, the curve was characterized by an IC_50_ of 27.58 ± 1.71 nM and a Hill coefficient of -0.85 ± 0.04 (mean ± SEM, *n* = 8, Table 2). Lotilaner antagonistic effect was characterized by a curve with an IC_50_ of 23.84 ± 1.87 nM and a Hill coefficient of -0.64 ± 0.03 (mean ± SEM, *n* = 16, Table 2). For the biologically inactive enantiomer, AHC-2229544, no inhibition of the GABA-induced current was observed up to 0.3 μM and some antagonism was observed at higher concentrations (Fig. 3b). The antagonism observed at the highest doses is most likely due to contaminating traces of lotilaner. The chiral purity of AHC-2229544 was determined at 99.65% (data not shown). As a consequence, at 10 μM of AHC-2229544, there is as much as 35 nM of lotilaner contamination, which corresponds to its IC_50_. The curve was characterized by an IC_50_ of 959.47 ± 268.44 nM and a Hill coefficient of -0.30 ± 0.03 (mean ± SEM, *n* = 8, Table 2). Additional file 4: Figure S2 shows typical cumulative dose response traces measured from oocytes expressing DmS-GABA and exposed to (a) dieldrin, (c) fipronil and (f) AHC-2229544.

**Table 2:** GABACl receptors response profiles to antagonists. IC_50_ in nM ± SEM obtained with a variety of compounds

Receptor	Antagonist	IC_50_ (nM)	n_H_	*N*
DmS-GABA	Dieldrin	4170 ± 920	-0.45 ± 0.05	9
DmS-GABA	Fipronil	27.58 ± 1.71	-0.85 ± 0.04	8
DmS-GABA	Lotilaner	23.84 ± 1.87	-0.64 ± 0.03	16
DmS-GABA	AHC-2229544	959.47 ± 268.44	-0.30 ± 0.03	8
DmR2-GABA	Dieldrin	> 10,000	-0.15 ± 0.03	6
DmR2-GABA	Fipronil	230.32 ± 27.29	-0.57 ± 0.04	7
DmR2-GABA	Lotilaner	38.25 ± 3.75	-0.62 ± 0.04	7
Ls-GABA1	Fipronil	164.85 ± 11.80	-0.78 ± 0.04	12
Ls-GABA1	Lotilaner	52.40 ± 4.54	-0.75 ± 0.04	8
Rm-GABA	Fipronil	25.56 ± 2.25	-0.56 ± 0.03	6
Rm-GABA	Lotilaner	36.79 ± 4.39	-0.47 ± 0.03	8
Cl-GABA_A_ α1β2γ2	Lotilaner	> 10,000	-0.09 ± 0.02	7

*Abbreviations*: Dm, *Drosophila melanogaster*; Ls, *Lepeophtheirus salmonis*; Rm, *Rhipicephalus microplus*; Cl, *Canis lupus familiaris.* The Hill coefficient (n_H_) is indicated as well as the number of oocytes evaluated (*N*)

**Fig. 4:**
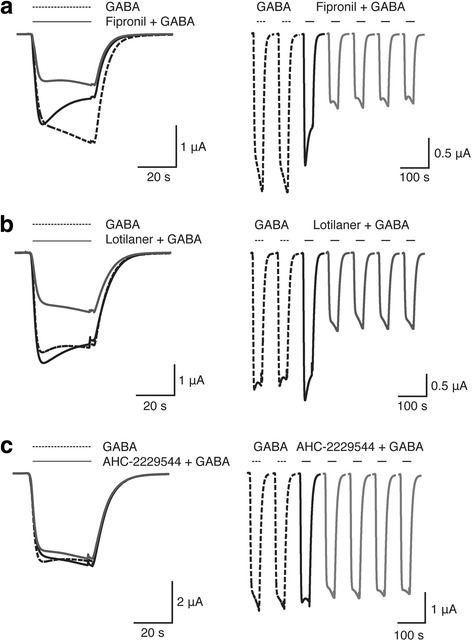
Co-application protocol on DmS-GABA. Current traces obtained from *X. laevis* oocytes expressing DmS-GABA receptors. The left panel shows superimposed traces recorded after the fifth exposure to 1 μM GABA (doted-line trace) and the first and second co-application (black and grey trace, respectively) with 100 nM (**a**) fipronil, (**b**) lotilaner, or (**c**) AHC-2229544 at 1 min interval. The doted-line traces show the fourth and fifths response to 1 μM GABA application. The right panel shows the forth and fifth GABA applications (doted-line) while the black and grey traces represent the first and the second to fifth co-application with 100 nM of (**a**) fipronil, (**b**) lotilaner, or (**c**) AHC-2229544, respectively. The bars indicate the time period of GABA application (interrupted line) or GABA co-applied with the compound (solid line)

We further characterized lotilaner blocking properties on oocytes expressing the DmS-GABA receptor and compared it to fipronil and AHC-2229544. We used a co-application protocol where the drugs were directly co-applied with GABA without any pre-incubation time, to assess the blocking effect on activated receptors. In those experiments, GABA was used at 1 μM corresponding to the EC_10_ to keep the channel desensitization to a minimum. After the first co-application with fipronil (Fig. 4a), the averaged normalized peak currents represented 78.26 ± 13.87% and went down to 35.53 ± 13.13% after the second one (mean ± SD, *n* = 16; Additional file 5: Figure S3). The averaged normalized tail currents where measured at 54.69 ± 12.63% and 35.73 ± 12.85% after first and second co-application respectively (mean ± SD, *n* = 16; Additional file 5: Figure S3). Fipronil clearly blocked the activated receptors by gradually suppressing the peak amplitude currents as well as by accelerating the current decay. When we co-applied lotilaner (Fig. 4b), the averaged normalized peak currents represented 132.80 ± 26.91% and went down to 72.29 ± 14.87% after the second one (mean ± SD, *n* = 22; Additional file 5: Figure S3). The averaged normalized tail currents where measured at 113.40 ± 19.43% and 78.31 ± 14.81% after first and second co-application respectively (mean ± SD, *n* = 22; Additional file 5: Figure S3). Interestingly, we observed for lotilaner a current potentiation at first co-application followed by a receptor blockade with the subsequent co-applications. The peak and tail currents were potentiated on average by > 30% and > 10%, respectively. However, the current decay was accelerated bringing the tail currents close to the values recorded with GABA only. The blocking effect of lotilaner became visible at the second co-application and remained stable with the next three drug applications. With AHC-2229544 (Fig. 4c), the averaged normalized peak currents represented 103.00 ± 7.31% and went down to 91.73 ± 8.09% after the second exposure (mean ± SD, *n* = 10; Additional file 5: Figure S3). The averaged normalized tail currents where measured at 100.8 ± 7.27% and 92.34 ± 7.64% after first and second co-application respectively (mean ± SD, *n* = 10; Additional file 5: Figure S3). As expected, AHC-2229544 produced no significant effect on the peaks neither on the tail currents. The slight decrease in both peaks and tails currents (< 10%) could be explained by a minor receptor desensitization or might be due to contaminating traces of the active enantiomer (see above for a detailed explanation).

### Lotilaner is a non-competitive antagonist of DmS-GABA receptor

**Fig. 5:**
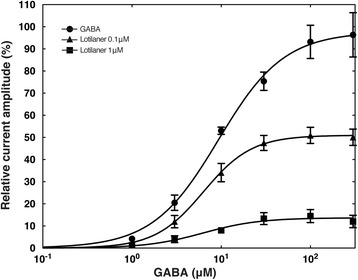
Lotilaner function as a non-competitive antagonist of DmS-GABA receptors. Concentration-response plots for GABA alone (black circle), GABA in the presence of 0.1 μM lotilaner (black triangle), and GABA in the presence of 1 μM lotilaner (black square). EC_50_ and R_max_ values were 9.29 ± 0.81 μM and 97.706 ± 2.45% (*n* = 5) for GABA, 6.33 ± 0.20 μM and 50.98 ± 0.47% (*n* = 5) in 0.1 μM lotilaner, and 6.27 ± 1.85 μM and 13.65 ± 1.12% (*n* = 4) in 1 μM lotilaner. Individual curves were standardized to an initial 100 μM GABA application and subsequently averaged. Mean ± SEM is shown

To elucidate the type of antagonism produced by lotilaner, we first applied 1, 3, 10, 30, 100 and 300 GABA (μM) in the absence of lotilaner, which served as the control. This was followed by a different set of experiments where we applied the same concentration of GABA in the presence of 0.1 and 1 μM lotilaner. The concentration-response relationships for GABA in the absence and presence of 0.1 and 1 μM lotilaner are shown in Fig. 5 and were fitted with the eq. 2. Individual curves were standardized to an initial 100 μM GABA application and subsequently averaged. The EC_50_ and R_max_ values were 9.29 ± 0.81 μM and 97.706 ± 2.45% (*n* = 5) for GABA alone, 6.33 ± 0.20 μM and 50.98 ± 0.47% (*n* = 5) in presence of 0.1 μM lotilaner, and 6.27 ± 1.85 μM and 13.65 ± 1.12% (*n* = 4) in presence of 1 μM lotilaner. Lotilaner showed a concentration-dependent significant reduction of R_max_ (t-test: *t*_(8)_ = 4.336, *P* = 0.0025 and *t*_(7)_ = 7.260, *P* = 0.0002 for 0.1 μM and 1 μM, respectively), with no change in EC_50_ (t-test: *t*_(8)_ = 0.9321, *P* = 0.3786 and *t*_(7)_ = 0.5933, *P* = 0.5716 for 0.1 μM and 1 μM, respectively), which are characteristics of non-competitive antagonism. These results indicate that lotilaner is a non-competitive antagonist of the DmS-GABA receptors.

### Lotilaner breaks resistance to dieldrin or fipronil

**Fig. 6:**
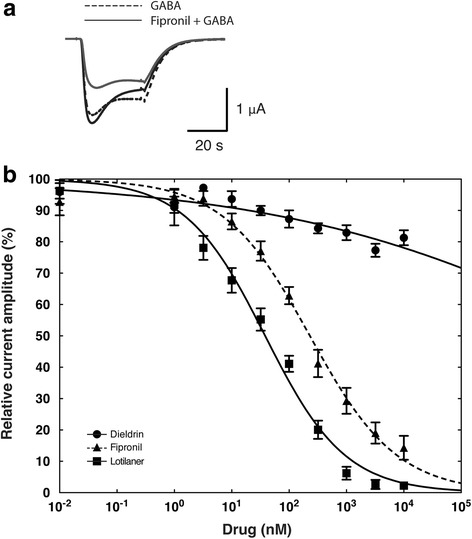
Lotilaner antagonism is not affected by mutation causing dieldrin and fipronil resistance. **a** Current traces obtained from a *X. laevis* oocyte expressing DmR2-GABA receptors. The interrupted trace shows the fifths response to 1 μM GABA application representing the baseline. The black and grey traces (first and second recording, respectively) have been obtained after oocyte exposure to 1 μM GABA co-applied with 100 nM fipronill. The bars indicate the time period of GABA application (interrupted line) or GABA co-applied with fipronil (solide line). **b** Averaged inhibition concentration-response curves measured for dieldrin (black circle), fipronil (black triangle, dashed line) and lotilaner (black square), obtained from oocytes expressing DmR2-GABA. Individual curves were standardized to the fitted maximal current amplitude and subsequently averaged. Mean ± SEM of experiments carried out with at least four oocytes from two batches each is shown

To determine if lotilaner could break resistance to dieldrin and fipronil, we used a mutant DmR2-GABA gene in which two amino acid substitutions (A301S and T350 M) were present. We used both, a co-application and a pre-application protocol as previously described for DmS-GABA. Figure 6a, shows superimposed traces recorded after the fifth exposure to 1 μM GABA (doted-line trace) and the first and second co-application (black and grey trace, respectively) with 100 nM fipronil at 1 min interval. After the first co-application with fipronil, the averaged normalized peak currents represented 96.78 ± 14.24% and went down to 61.4 ± 11.22% after the second one (mean ± SD, *n* = 17; Additional file 5: Figure S3). The averaged normalized tail currents where measured at 83.74 ± 10.10% and 62.26 ± 11.18% after first and second co-application respectively (mean ± SD, *n* = 17; Additional file 5: Figure S3). Compared to DmS-GABA (Fig. 5a), the first application of fipronil had almost no effect on the peak amplitude currents and the current decay was substantially reduced (Fig. 6a, black trace). After the second exposure to fipronil a block was visible but not as pronounced as with DmS-GABA (Fig. 6a, grey trace). The peak and tail currents were 1.24 and 1.73 time bigger after the first and 1.53 and 1.74 times bigger than DmS-GABA after the second application respectively.

Figure 6b shows averaged inhibitory concentration-response curves for DmR2-GABA challenged with dieldrin (open circles), fipronil (closed circles) or lotilaner (closed squares). The dieldrin curve was characterized by an IC_50_ > 10 μM and a Hill coefficient of -0.15 ± 0.03 (mean ± SEM, *n* = 6, Table 2), the fipronil curve was characterized by an IC_50_ of 230.32 ± 27.29 nM and a Hill coefficient of -0.57 ± 0.04 (mean ± SEM, *n* = 7, Table 2) and the lotilaner curve was characterized by an IC_50_ of 38.25 ± 3.75 nM and a Hill coefficient of -0.62 ± 0.04 (mean ± SEM, *n* = 7, Table 2). The resistance factors compared to DmS-GABA are > 12,000 for dieldrin, 8.35 for fipronil and 1.60 for lotilaner. The mutant channel was totally resistant to dieldrin and partially to fipronil with inhibitory concentration-response curves and IC_50_ clearly shifted to the right. In contrast, the inhibitory concentration-response curves measured on DmR2-GABA with lotilaner was almost superimposed to the one measured on DmS-GABA. Additional file 4: Figure S2 shows typical cumulative dose response traces measured from oocytes expressing DmR2-GABA and challenged with dieldrin (b), fipronil (d) or lotilaner (e). Our results suggest that no cross-resistance would be expected between lotilaner and dieldrin or fipronil.

### Lotilaner is a potent antagonist of invertebrate GABACl receptors

**Fig. 7:**
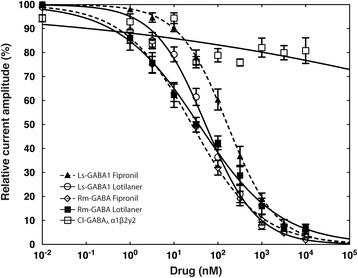
Lotilaner is a potent antagonist of invertebrate GABACl receptors. Averaged inhibition concentration-response curves for lotilaner and fipronil measured on oocytes expressing Ls-GABA1 (white circle and black triangle, dashed line) or Rm-GABA (black square and white diamond, dashed line) as well as on Cl-GABA_A_ α1β2γ2 (white square) for lotilaner. Individual curves were standardized to the fitted maximal current amplitude and subsequently averaged. Mean ± SEM of experiments carried out with at least four oocytes from two batches each is shown

To further assess the antagonistic effects of lotilaner, we tested it on oocytes expressing the crustacean Ls-GABA1 and the acari Rm-GABA receptors. Figure 7 shows averaged inhibitory concentration-response curves for Ls-GABA1 and Rm-GABA upon lotilaner or fipronil exposure. The Ls-GABA1 receptors were found to be more sensitive to the antagonistic effects of lotilaner (open circles) than fipronil (closed triangles). The lotilaner curve was characterized by an IC_50_ of 52.40 ± 4.54 nM and a Hill coefficient of -0.75 ± 0.04 (mean ± SEM, *n* = 8, Table 2) while the fipronil curve was characterized by an IC_50_ of 164.85 ± 11.80 nM and a Hill coefficient of -0.78 ± 0.04 (mean ± SEM, *n* = 12, Table 2). The Rm-GABA receptors were also strongly antagonized by lotilaner (closed squares) and fipronil (open diamond). The lotilaner curve was characterized by an IC_50_ of 36.79 ± 4.39 nM and a Hill coefficient of -0.47 ± 0.03 (mean ± SEM, *n* = 8, Table 2) while the fipronil curve was characterized by an IC_50_ of 25.56 ± 2.25 nM and a Hill coefficient of -0.56 ± 0.03 (mean ± SEM, *n* = 6, Table 2). Finally, we tested lotilaner on a dog GABACl (Cl-GABA_A_ α1β2γ2) to address the specificity of lotilaner towards invertebrate GABACls. In contrast to invertebrates, vertebrate GABACls are heteropentamers made of more than one subunit. No obvious inhibitory effect was found even at the highest dose tested (10 μM; Fig. 7 and Table 2). Additional file 6: Figure S4 shows typical cumulative dose response traces measured from oocytes expressing Ls-GABA1 (A and B), Rm-GABA (C and D) and Cl-GABA_A_ α1β2γ2 (E) upon antagonist exposure.

## Discussion

The first step of our investigations on the molecular mode of action of lotilaner comprised the identification, full length cDNA cloning and demonstration of functionality of the putative target genes from insects (*D. melanogaster*, DmS-GABA), ticks (*R. microplus*, Rm-GABA), crustaceans (*L. salmonis*, Ls-GABA1) and mammals (*C. lupus familiaris*, Cl-GABA_A_ α1β2γ2), to build the basis for parasites and host on-target studies (Additional file 3 Figure S1a-f). To complement our molecular investigations we generated a *D. melanogaster* mutant GABA channel (DmR2-GABA) by integrating two amino acid substitutions in the M2 and M3 respectively (A301S; T350 M) that have previously been shown to confer dieldrin and fipronil resistance to investigate their effect on lotilaner potency (Fig. 2). We concentrated our efforts on GABACls only since they have been shown to act as the primary target for the isoxazoline compounds [16].

A total of 5 GABACls were cloned and expressed in *Xenopus* oocytes to perform comparative determinations of agonist EC_50_ values and antagonist IC_50_ values. Functional expression and TEVC studies on DmS- and DmR2-GABA cRNA injected into *Xenopus* oocytes demonstrated that both gene products acted as GABA channel. In the case of DmR2-GABA, importantly, the GABA EC_50_ values did not differ significantly from the wild type DmS-GABA receptor (12.43 μM *vs* 10.52 μM, Table 1) and had no impact on the current shapes or their kinetic. Those values are in the same range as previously reported values from *Xenopus* oocyte voltage-clamp electrophysiology made on DmRDLs corresponding to the *ac* splice variant [7, 19, 22–24]. In addition, we cloned the gene encoding *R. microplus* GABA receptor based on DNA sequences published in GenBank (accession number GQ398111.1). This tick species is considered to be the most important tick parasite of livestock in the world. *Rhipicephalus microplus* mainly infests cattle, deer and buffalo, but it can also be found on horses, goats, sheep, donkeys, dogs, pigs and some wild mammals. The Rm-GABA translated ORF used in this study was 99% identical to the protein sequence described by Gassel et al. [16] (GenBank: AHE41094.1) but two lysines replaced by two arginines in the extracellular loop of the subunit. We confirmed that Rm-GABA expression product was an RDL receptor with an EC_50_ value (49.96 μM, Table 1) in the same range to the one reported by Gassel et al. with RmRDL (9.8 μM) expressed in HEK293 cells [16]. To further assess the spectrum of activity of lotilaner, we cloned and functionally expressed for the first time a GABACl from a crustacean (Ls-GABA1), *L. salmonis*, an ectoparasite of the Atlantic salmon, *Salmo salar*. Sea lice (*L. salmonis* and *Caligus* spp.) are the major pathogens affecting the global salmon farming industry and have a significant economic impact in many areas. Prevention and control strategies are required to eliminate or minimize the disease but emerging resistance to most used products increases the necessity to develop new treatment methods (biological, prophylactic and new medicine) and tools to avoid increased losses due to sea lice and to ensure a sustainable salmon farming industry in the future [25–27]. For Ls-GABA1, bioinformatic analysis predicted the presence of a signal sequence for import into the endoplasmic reticulum as well as four transmembrane helices, with the arrangement of extracellular and intracellular domains in agreement with the known architecture of ligand-gated ion channels (data not shown). Ls-GABA1 was robustly expressed in *Xenopus* oocytes with > 5 μA currents record upon GABA application. Nevertheless, its sensitivity to GABA was the lowest one (EC_50_ = 392.54 μM, Table 1) compared to the other GABACls used in this study. Finally, we wanted to address the specificity of lotilaner towards invertebrates GABACls compared to a vertebrate homologous receptor. For this purpose, we reconstituted the dog α1β2γ2 GABA_A_ receptor subtype (α1β2γ2), which is the most abundant receptor subtype in the vertebrate brain [28]. It is well documented that equal ratios of cRNA coding for α1-, β2-, and γ2-subunits injected in *Xenopus* oocytes or cDNA coding for α1, β2 and γ2 co-transfected in HEK293 cells results in both cases in a mixed population of α1β2 and α1β2γ2 receptors [29, 30]. To ensure that γ2 subunits are integrated into the heteropentamers, we have injected 5 times more γ2 than α1 or β2 cRNA but we cannot exclude that α1β2 subtypes were also expressed. Diazepam, a positive allosteric modulator of α1β2γ2 but not α1β2 was used to demonstrate the presence of GABACls containing the γ2 subunit [31]. Cl-GABA_A_ α1β2γ2 current traces were characterized by a rapid desensitization phase upon GABA stimulation followed by a steady-state current in a second phase. This receptor proved to be the most sensitive to GABA with an EC_50_ < 2 μM, a value significantly lower than values previously published with human α1β2γ2 GABACl. For instance, Minier & Sigel [31] reported an EC_50_ value for human α1β2γ2 and α1β2 GABACl of 41 ± 18 μM and 8 ± 2.2 μM, respectively.

In a second step in this study, we used our insect subunits to assess inhibitory actions by insecticides, including the novel isoxazoline compound lotilaner (Fig. 1). We started our investigations with DmS-GABA, a wild-type GABACl subunits from *D. melanogaster*, also known as the RDL subunit in comparison with a mutant channel made of the DmR2-GABA subunit (Fig. 2). The most prominent representative of the cyclodiene group of insecticides, dieldrin, showed an inhibitory action on DmS-GABA with an IC_50_ value of 4.17 μM in agreement with previously reported values [16] while, as expected, no inhibition was seen on DmR2-GABA (Figs. 3 and 6 and Table 2). Fipronil (Fig. 1) is a phenylpyrazole insecticide and acaricide introduced on the market in 1993 and used in crop protection and veterinary medicine. Many studies have shown that this compound could block both GABACls and GluCls [32–34]. In our experiments, fipronil proved to be an effective inhibitor of DmS-GABA (IC_50_ = 27.58 nM), but the resistance mutations present in DmR2-GABA did lead to a significant loss of potency by a factor of 8 (IC_50_ = 230.32 nM, Figs. 3 and 6 and Table 1). This was in a similar range than previously reported with the *D. melanogaster* S_302_ form (18-fold; [16]). Isoxazolines have emerged recently as a novel class of parasiticides targeting GABACls and GluCls of insects and acari [16, 21, 35, 36]. The experiments conducted in this study have shown that the isoxazoline drug lotilaner inhibits the *D. melanogaster* GABACl in the low nanomolar IC_50_ range. Only a minor, statistically not significant difference was measured between the DmS- and DmR2-GABA channel version (IC_50_ values of 23.84 nM and 38.25 nM, respectively; Table 2), suggesting that lotilaner is not affected by the dieldrin and fipronil resistance mutations. Similar results with isoxazoline compounds have been published earlier on *Musca domestica* [21], *D. melanogaster* [35] and *C. felis* GABACl with RDL mutations [16]. One possible explanation is that isoxazoline analogues might circumvent cross-resistance by addressing a distinct new binding pocket in the chloride channels and is as a consequence not negatively impacted by the dieldrin or fipronil resistance mutations. Additional experiments would be required to support this hypothesis.

The IC_50_ values we measured with lotilaner on DmS-GABA were approximately 10-fold higher than the one reported by Gassel et al. [16] measured with a membrane potential dye assay set-up. While this difference could be attributed to the compound potency itself, there is the possibility that voltage-clamp electrophysiology readouts of *Xenopus* GABACl expression systems require higher concentrations of some antagonists/agonist compared to the membrane potential dye assays in cell culture [16]. Finally, we have shown that the antagonistic effect on GABACls was enantiomer-dependent: in contrast to lotilaner, there was no significant inhibitory action of AHC-2229544 on DmS-GABA (Figs. 3b and 4c).

To complement our investigations, we tested the effect of fipronil, lotilaner and AHC-2229544 using a co-application protocol with no preliminary exposure of the channels to the drug. Surprisingly, we observed a significant GABA current potentiation after the first co-application, followed by a current inhibition during the subsequent co-applications (Fig. 4). With a co-application protocol, the antagonist is applied to already open channels while with a pre-application protocol channels are in a closed state. It has been suggested that the second generation non-competitive antagonists (NCA-II, e.g. isoxazoline) target site is localized in a pore between the T9’ to S15’ region, an interstitial subunit region [37]. The same authors hypothesized that NCA-II might enter into the pore and then migrates to the interstitial region or *vice versa* where they could trigger channel closure or stabilize the closed state. With a pre-application, lotilaner might have the time to migrate within the pore of the GABACl to its final location stabilising it in a closed state. Without a pre-application, lotilaner might stay within a primary location where it destabilizes the open state allowing a higher amount of ions to flow through the GABACl pore. This hypothesis has so far not been experimentally addressed.

As a third step, by taking advantage of the *Xenopus* oocyte expression system, we have demonstrated using TEVC electrophysiology that lotilaner acts as a non-competitive antagonist of the DmS-GABA. Despite a strong current inhibition, no change in the EC_50_ for GABA was measured in the presence of lotilaner, which is characteristic for a non-competitive antagonism (Fig. 5).

In a last step, we used our crustacean, acarine and mammalian subunits to assess inhibitory actions by fipronil (only for the crustacean GABACl) and lotilaner. Fipronil inhibited the crustacean ion channel (Ls-GABA1) with an IC_50_ of 164.85 nM (Fig. 7 and Table 2), which is in agreement with in vivo observations where pyriprole, belonging to the same class as fipronil, was shown to act as a potent inhibitor of *L. salmonis* larval development [38]. Lotilaner proved to be a potent inhibitor of Ls-GABA1 as a 3-fold lower IC_50_ value compared to fipronil was measured (Fig. 7 and Table 2). This result is in agreement with previously published values where a higher inhibitor potency of fluralaner over fipronil was measured on *M. domestica* (5-fold; [21]) and *R. microplus* GABACls (5-fold; [16]). Consequently, for the first time, we demonstrate that isoxazoline compounds have a potent antagonistic effect on crustacean GABACls. Nevertheless, lotilaner innocuity towards fish (from farms or from the wild) and other crustaceans remains to be proven. The tick GABACl (Rm-GABA) receptors were also strongly antagonized by lotilaner and fipronil but the measured IC_50_ (36.79 nM and 25.56 nM, respectively) are approximately 20- and 3-fold higher respectively than the one reported by Gassel et al. [16]. As already mentioned, this difference might be due to the experimental set-up, or in the case of the isoxazolines, to the intrinsic potency of the tested molecules. Finally, we show that the activity of lotilaner seems to be arthropod-specific, since no activity on a dog GABA_A_ receptor was observed up to a concentration of 10 μM (Fig. 7). To address the specificity of lotilaner towards invertebrates GABACls compared to vertebrate homologous receptor, additional GABACls, for example, from cats, rabbits or other dog breeds will have to be tested.

## Conclusions

In summary, our study demonstrates that the novel isoxazoline parasiticide lotilaner acts as a potent non-competitive antagonist of GABACls from insects (fly), Acari (tick) and crustaceans (sea lice) while it shows no effect on dog GABA_A_ receptors up to a concentration of 10 μM (subtype α1β2γ2). No cross-resistance with dieldrin or fipronil resistance mutations was detected, suggesting that lotilaner likely binds to a site a least partly different from the one bound by known blockers in GABACl. This work enhances our understanding of lotilaner mode of action in ectoparasites and additionally could support the development of genetic markers for the early detection of resistant genotypes, which may develop in the future.

## Additional files

Additional file 1: Table S1. Primers used for PRC amplification of *Canis lupus familiaris* and *Lepeophteirus salmonis* GABACl subunits. (DOC 36 kb)

Additional file 2: French translation of the Abstract. (PDF 61 kb)

Additional file 3: Figure S1. Sample dose response curves for GABA. The bars indicate the time period of GABA application (20 s). GABA concentrations in μM are indicated above the bars. Traces obtained from an oocyte expressing (**a**) DmS-GABA, (**b**) DmR2-GABA, (**c**) Ls-GABA1, (**d**) Rm-GABA, (**e**) Cl-GABA_A_ α1β2γ2. (**f**) Averaged GABA concentration-response curves measured DmS-GABA (black circle), DmR2-GABA (black triangle), Rm-GABA (black square), Ls-GABA1 (white square) and Cl-GABA_A_ α1β2γ2 (white triangle). Individual curves were standardized to the fitted maximal current amplitude and subsequently averaged. Mean ± SEM of experiments carried out with at least four oocytes from two batches each is shown. (TIFF 755 kb)

Additional file 4: Figure S2. Example of cumulative dose response curves measured on oocytes expressing DmS- or DmR2-GABA. The bars indicate the time period of GABA application (20 s). The grey triangle represents the gradual exposure to a given compound with the respective concentration in μM indicated above. Traces obtained from an oocyte expressing DmS- and DmR2-GABA receptors and exposed to dieldrin (**a**, **b**), fipronil (**c**, **d**), lotilaner (**e**; DmR2-GABA only) and AHC-2229544 (**f**; DmS-GABA only). GABA was used at a concentration of 10 μM. (TIFF 767 kb)

Additional file 5: Figure S3. Lotilaner is not affected by dieldrin and fipronil resistance mutations. Box plots obtained from oocytes expressing the DmS- (shadow boxes) or DmR2-GABA (white boxes). Currents were measured at the peak (highest current) or at the tail (30 s after the co-application) of the trace after exposure to lotilaner (black boxes), fipronil (red boxes) or AHC-2229544 (blue boxes). Outliers are shown as small black circles, the medium by a +. The boxes show the lower and upper quartile with the median represented by a line. The whiskers show the 10 and 90 percentile, respectively. Values above the interrupted line represent a current stimulation while values below denote a current blockade. (TIFF 1116 kb)

Additional file 6: Figure S4. Example of cumulative dose response curves. The bars indicate the time period of GABA application (20 s). The grey triangle represents the gradual exposure to a given compound with the respective concentration in μM indicated above. Traces obtained from an oocyte expressing Ls-GABA1 receptors and exposed to (**a**) fipronil (dashed-line) and (**b**) lotilaner. Traces obtained from an oocyte expressing Rm-GABA receptors and exposed to (**c**) fipronil (dashed-line) and (**d**) lotilaner. Traces obtained from an oocyte expressing Cl-GABA_A_ α1β2γ2 receptors and exposed to (**e**) lotilaner. GABA was used at a concentration of 500 μM (**a**, **b**), 50 μM (**c**, **d**) and 2 μM (**e**). (TIFF 737 kb)

## Abbreviations

SD: standard deviation
SEM: standard error of the mean
