# Supplementary material for: The novel isoxazoline ectoparasiticide lotilaner (Credelio™): a non-competitive antagonist specific to invertebrates γ-aminobutyric acid-gated chloride channels (GABACls)
Source: Parasit Vectors. 2017 Nov 1;10:530. doi: 10.1186/s13071-017-2470-4 (PMC5664438; doi:10.1186/s13071-017-2470-4)
Supplement: Supplementary file 1 — Primers used for PRC amplification of Canis lupus familiaris and Lepeophteirus salmonis GABACl subunits. (DOC 36 kb) [file 13071_2017_2470_MOESM1_ESM.doc]

**Additional file 1: Table S1.** Primers used for PCR amplification of *Canis lupus familiaris* and *Lepeophtheirus salmonis* GABACl subunits.

| **Primer name** | **Sequence 5’ → 3’** |
| --- | --- |
| *For PCR on cDNA* |  |
| **NheI**_Cl-GABAa1_F1 | GGCG**GCTAGC**CGCGATGAAGAAAAGTCTGG |
| **XhoI**_Cl-GABAa1_R1 | GGCG**CTCGAG**CCAGTGCAGAGGACTGAACA |
| **NheI**_Cl-GABAb2_F1 | GGCG**GCTAGC**CCCATCAAAAACTAAAGGGATG |
| **XhoI**_Cl-GABAb2_R1 | GGCG**CTCGAG**CCCATGGGAGGCTATGTTTTA |
| **NheI**_Cl-GABAg2_F1 | GGCG**GCTAGC**CCGAGAGACTAGAGGCAGAGG |
| **XhoI**_Cl-GABAg2_R1 | GGCG**CTCGAG**CCTCTTTCACAGGTAAAGGTAGGA |
| Ls-GABA1_F2 | TGCGAGATTCAATTTGTTCG |
| Ls-GABA1_F3 | GTCGGTCTCGGTGTGACAA |
| Ls-GABA1_R6 | AATTCAGACCGTCGTTCCAC |
| Ls-GABA1_R7 | GTAGATCCATTGGGCACGAA |
| **NheI**_Ls-GABA1-F1 | GGCG**GCTAGC**CAAACATGAATAATCCCAGTGA |
| **SpeI**_Ls-GABA1-R1 | GGCG**ACTAGC**TTTGTGTATTTTAGTCCGGGTGT |
|  |  |
