# Supplementary material for: The novel isoxazoline ectoparasiticide lotilaner (Credelio™): a non-competitive antagonist specific to invertebrates γ-aminobutyric acid-gated chloride channels (GABACls)
Source: Parasit Vectors. 2017 Nov 1;10:530. doi: 10.1186/s13071-017-2470-4 (PMC5664438; doi:10.1186/s13071-017-2470-4)
Supplement: Supplementary file 2 — French translation of the Abstract. (PDF 61 kb) [file 13071_2017_2470_MOESM2_ESM.pdf]

Le lotilaner (Credelio™), un nouvel antiparasitaire externe de la classe des isoxazolines, est un inhibiteur non compétitif spécifique des canaux chlorure des récepteurs de l'acide γ-aminobutyrique (GABACl) des invertébrés

Lucien Rufener[1]*, Vanessa Danelli[1], Daniel Bertrand[2] et Heinz Sager[1]

[1]Elanco Santé Animale, Mattenstrasse 24a, 4058 Bâle, Suisse
[2]HiQScreen Sàrl, Route de Compois 6, CH-1222 Vésenaz, Suisse

*Correspondance : lucien_rufener@hotmail.com

Adresse électronique :
lucien_rufener@hotmail.com; vanessadanelli@hotmail.com; daniel.bertrand@hiqscreen.com
heinz.sager@elanco.com

Résumé

Contexte : Les isoxazolines, qui constituent une nouvelle classe d'antiparasitaires, sont de puissants inhibiteurs des canaux chlorure GABA (acide γ-aminobutyrique) dépendants (GABACl) et, dans une moindre mesure, des canaux chlorure glutamate dépendants (GluCl). Le lotilaner (Credelio™), un nouveau représentant de cette classe chimique, fait actuellement l'objet d'études en raison de ses remarquables propriétés antiparasitaires.

Méthodes: Lors de cette étude, le mode d'action moléculaire et la pharmacologie du lotilaner a été exploré. Nous avons réussi l'identification génique, le clonage de l'ADNc et l'expression fonctionnelle dans des ovocytes de xénope des GABACl de *Drosophila melanogaster* (type sauvage et formes résistantes à la dieldrine ou au fipronil), *Lepeophtheirus salmonis* (un crustacé copépode parasite du saumon), *Rhipicephalus microplus* et *Canis lupus familiaris*. Une technique d'électrophysiologie automatisée par voltage-clamp en doubles électrodes a été utilisée sur des ovocytes de xénope pour évaluer la fonctionnalité des GABACl et comparer l'inhibition des canaux ioniques par le lotilaner par rapport aux insecticides connus pour cibler les GABACl.

Résultats : Lors de ces tests, nous avons démontré que le lotilaner est un puissant inhibiteur non compétitif des GABACl des insectes (mouche). L'absence de détection de résistance croisée avec les mutations de résistance à la dieldrine ou au fipronil suggère que le lotilaner pourrait se fixer sur un site au moins en partie différent du site de liaison des inhibiteurs connus des GABACl. Lors d'expériences de co-application, nous avons observé que l'inhibition exercée par le lotilaner

est significativement différente de celle, classique, du fipronil qui bloque l'ouverture des canaux. Enfin, nous avons, pour la première fois, confirmé que les agents de la classe des isoxazolines sont non seulement de puissants inhibiteurs des GABACl d'acariens (tiques), mais aussi de crustacés (poux du poisson), tandis qu'aucune activité sur un récepteur $GABA_A$ canin n'a été observée.

Conclusions : Dans leur ensemble, ces résultats montrent que le lotilaner est un inhibiteur non compétitif spécifique des canaux chlorure GABACl des invertébrés. Ils nous permettent de mieux comprendre le mode d'action de ce nouvel antiparasitaire externe.
