# Supplementary figures and images for: The novel isoxazoline ectoparasiticide lotilaner (Credelio™): a non-competitive antagonist specific to invertebrates γ-aminobutyric acid-gated chloride channels (GABACls)
Source: Parasit Vectors. 2017 Nov 1;10:530. doi: 10.1186/s13071-017-2470-4 (PMC5664438; doi:10.1186/s13071-017-2470-4)

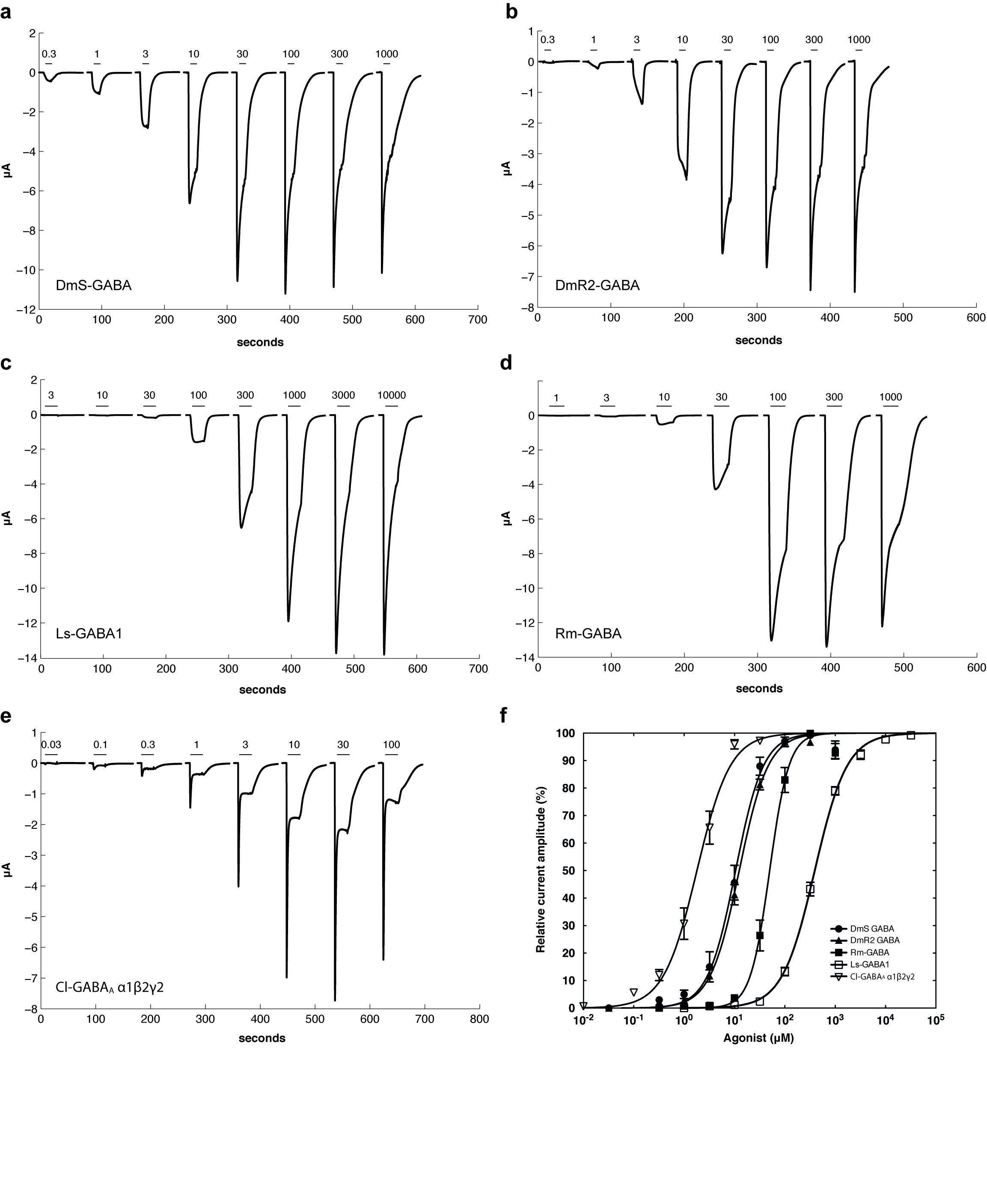

Supplement: Supplementary file 3 — Sample dose response curves for GABA. The bars indicate the time period of GABA application (20 s). GABA concentrations in μM are indicated above the bars. Traces obtained from an oocyte expressing (a) DmS-GABA, (b) DmR2-GABA, (c) Ls-GABA1, (d) Rm-GABA, (e) Cl-GABAA α1β2γ2. (f) Averaged GABA concentration-response curves measured DmS-GABA (black circle), DmR2-GABA (black triangle), Rm-GABA (black square), Ls-GABA1 (white square) and Cl-GABAA α1β2γ2 (white triangle). Individual curves were standardized to the fitted maximal current amplitude and subsequently averaged. Mean ± SEM of experiments carried out with at least four oocytes from two batches each is shown. (TIFF 755 kb) [file 13071_2017_2470_MOESM3_ESM.tif]

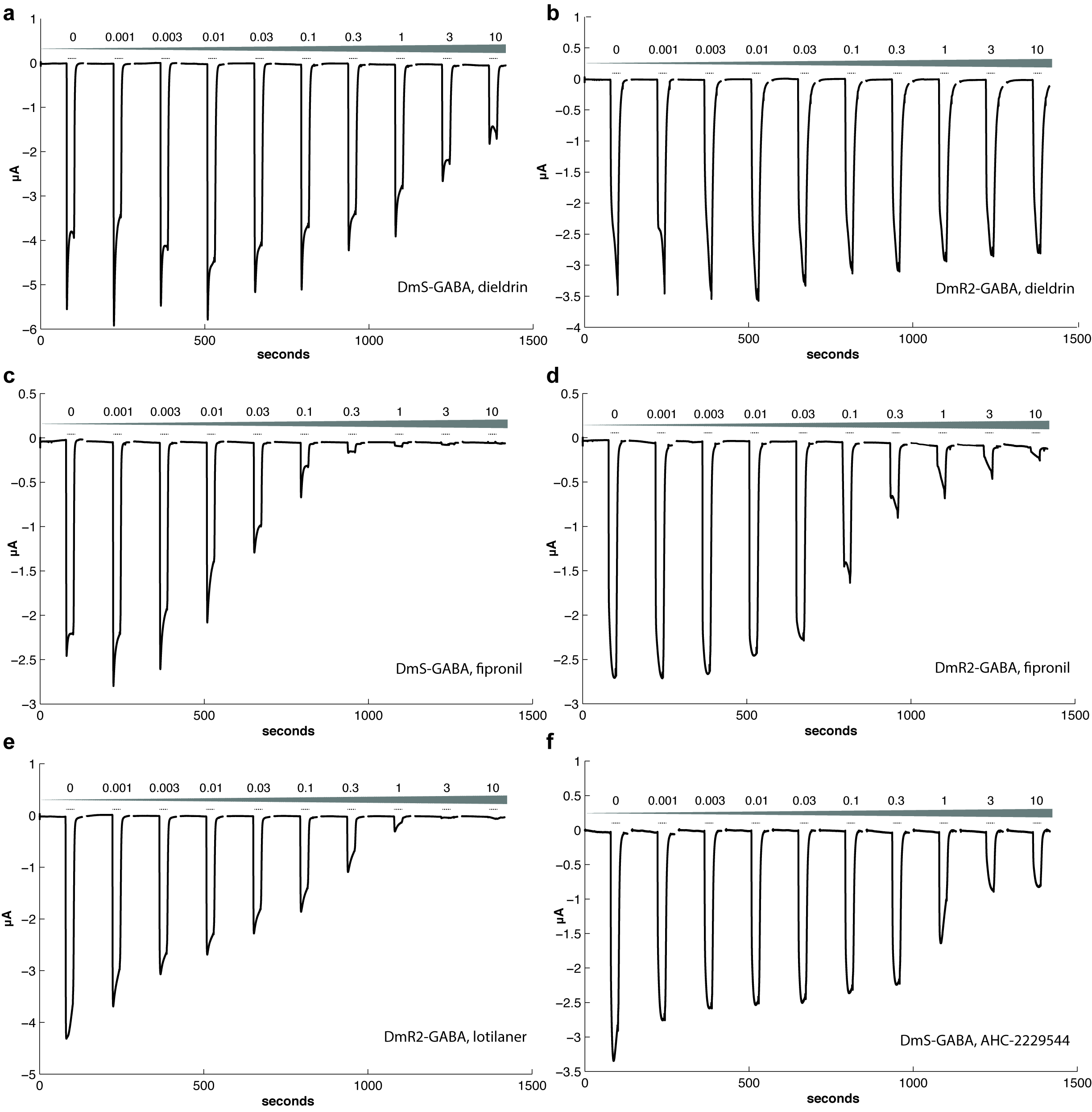

Supplement: Supplementary file 4 — Example of cumulative dose response curves measured on oocytes expressing DmS- or DmR2-GABA. The bars indicate the time period of GABA application (20 s). The grey triangle represents the gradual exposure to a given compound with the respective concentration in μM indicated above. Traces obtained from an oocyte expressing DmS- and DmR2-GABA receptors and exposed to dieldrin (a, b), fipronil (c, d), lotilaner (e; DmR2-GABA only) and AHC-2229544 (f; DmS-GABA only). GABA was used at a concentration of 10 μM. (TIFF 767 kb) [file 13071_2017_2470_MOESM4_ESM.tif]

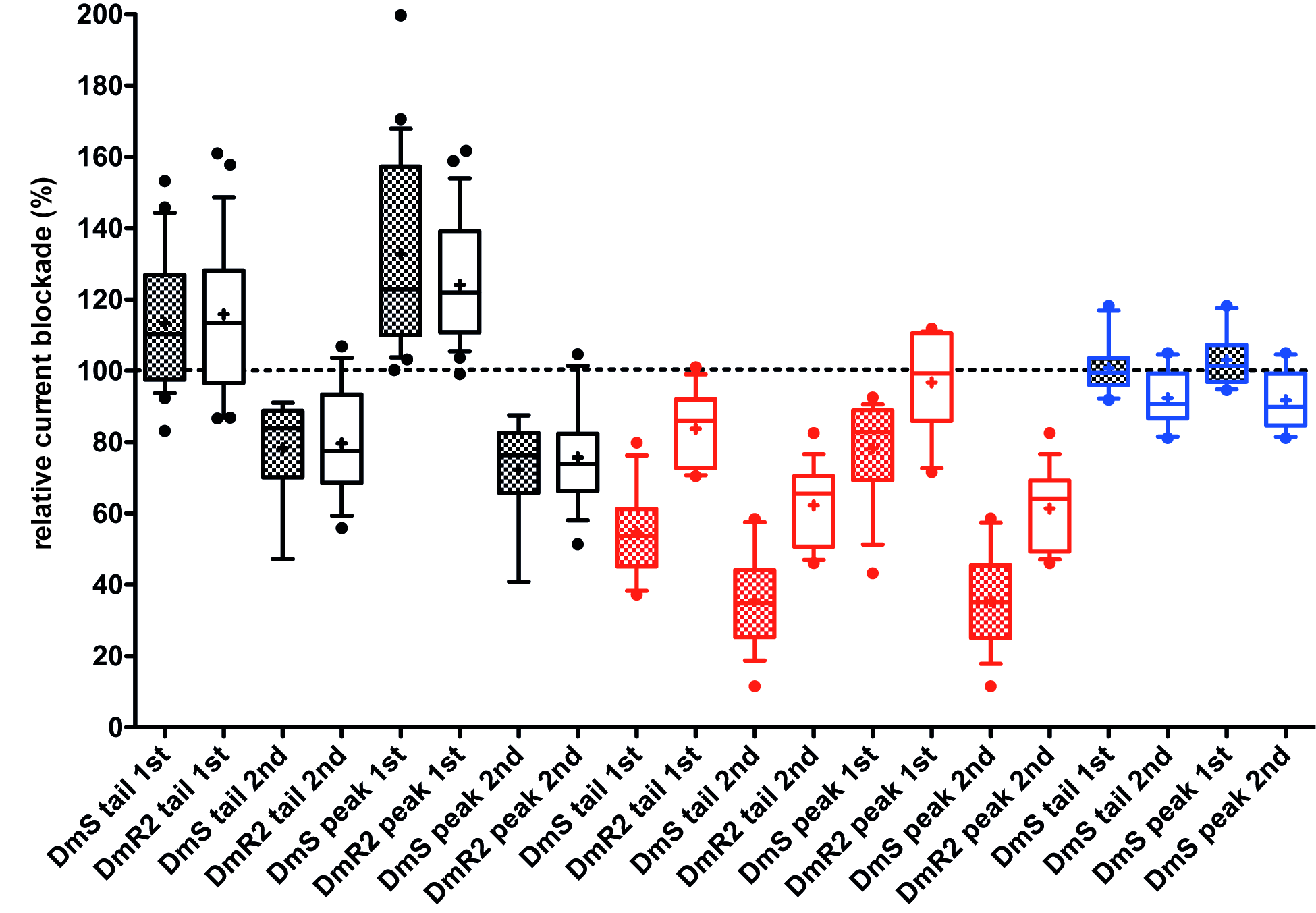

Supplement: Supplementary file 5 — Lotilaner is not affected by dieldrin and fipronil resistance mutations. Box plots obtained from oocytes expressing the DmS- (shadow boxes) or DmR2-GABA (white boxes). Currents were measured at the peak (highest current) or at the tail (30 s after the co-application) of the trace after exposure to lotilaner (black boxes), fipronil (red boxes) or AHC-2229544 (blue boxes). Outliers are shown as small black circles, the medium by a +. The boxes show the lower and upper quartile with the median represented by a line. The whiskers show the 10 and 90 percentile, respectively. Values above the interrupted line represent a current stimulation while values below denote a current blockade. (TIFF 1116 kb) [file 13071_2017_2470_MOESM5_ESM.tif]

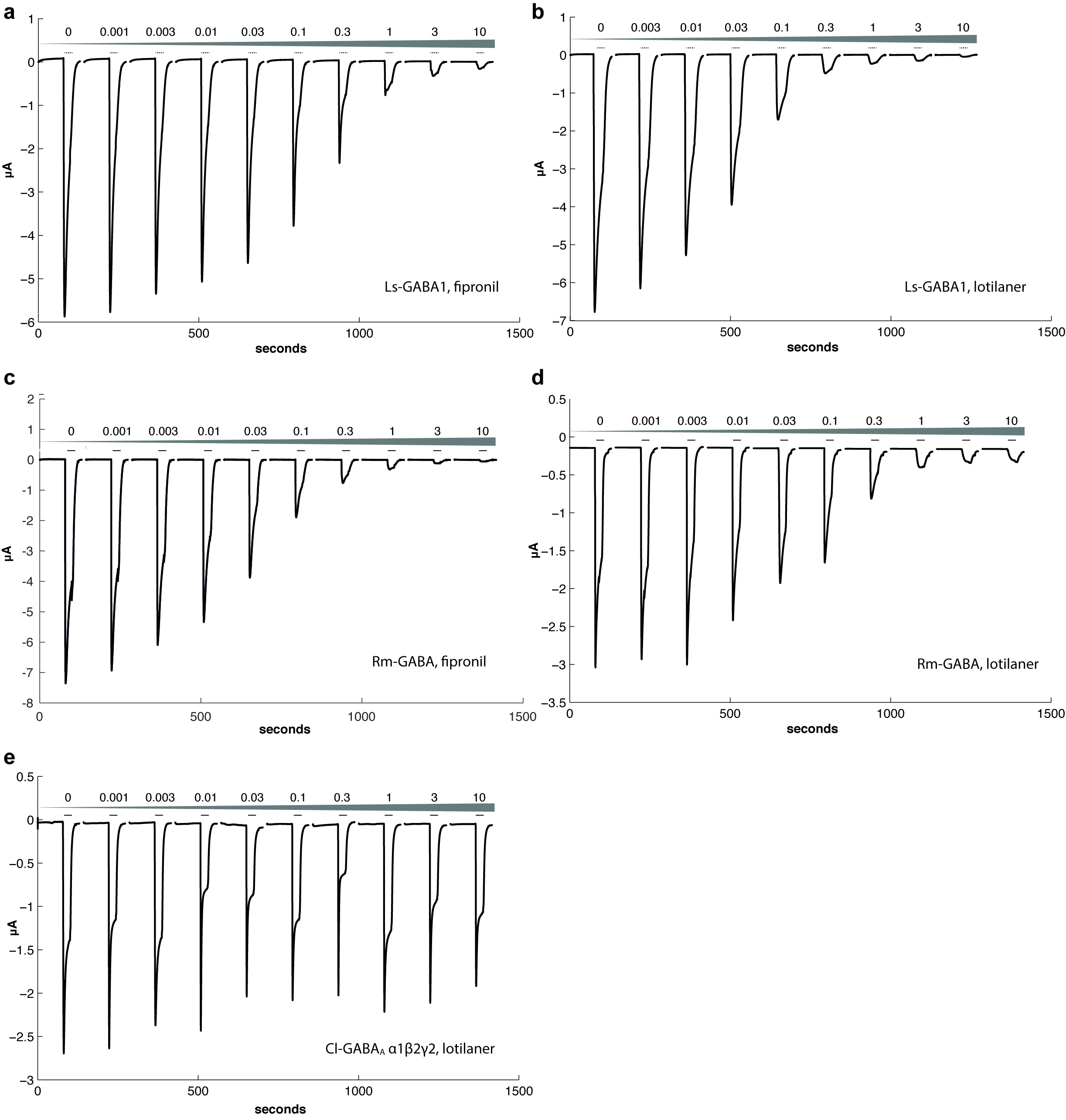

Supplement: Supplementary file 6 — Example of cumulative dose response curves. The bars indicate the time period of GABA application (20 s). The grey triangle represents the gradual exposure to a given compound with the respective concentration in μM indicated above. Traces obtained from an oocyte expressing Ls-GABA1 receptors and exposed to (a) fipronil (dashed-line) and (b) lotilaner. Traces obtained from an oocyte expressing Rm-GABA receptors and exposed to (c) fipronil (dashed-line) and (d) lotilaner. Traces obtained from an oocyte expressing Cl-GABAA α1β2γ2 receptors and exposed to (e) lotilaner. GABA was used at a concentration of 500 μM (a, b), 50 μM (c, d) and 2 μM (e). (TIFF 737 kb) [file 13071_2017_2470_MOESM6_ESM.tif]
